# The Shishu Pushti Trial–Extended Peer Counseling for Improving Feeding Practices and Reducing Undernutrition in Children Aged 0-48 Months in Urban Bangladesh: Protocol for a Cluster-Randomized Controlled Trial

**DOI:** 10.2196/31475

**Published:** 2022-02-07

**Authors:** Seema Mihrshahi, Gulshan Ara, Mansura Khanam, Sabrina Rasheed, Kingsley Emwinyore Agho, AKM Iqbal Kabir, S K Roy, Rukhsana Haider, Jena Derakhshani Hamadani, Fahmida Tofail, Ashraful Alam, Michael J Dibley

**Affiliations:** 1 Department of Health Sciences Faculty of Medicine, Health and Human Sciences Macquarie University Macquarie Park Australia; 2 Sydney School of Public Health The University of Sydney Sydney Australia; 3 Nutrition and Clinical Services Division International Centre for Diarrhoeal Disease Research, Bangladesh Dhaka Bangladesh; 4 School of Health Sciences Western Sydney University Campbelltown Australia; 5 Projahnmo Research Foundation Dhaka Bangladesh; 6 Bangladesh Breastfeeding Foundation Institute of Public Health Dhaka Bangladesh; 7 Training and Assistance for Health and Nutrition Foundation Dhaka Bangladesh

**Keywords:** child stunting, prevention, nutrition behavior change, breastfeeding: infant and young child feeding, peer counseling, child development

## Abstract

**Background:**

The aim of this study is to assess if peer counseling of women improves breastfeeding, complementary feeding practices, and child growth, and thus reduces the prevalence of undernutrition in children up to 4 years of age.

**Objective:**

Lack of exclusive breastfeeding and inappropriate complementary feeding are critical factors in reducing child undernutrition, morbidity, and mortality. There are reported trials of peer counseling to improve breastfeeding; however, they did not examine the efficacy of peer counseling to improve complementary feeding or the long-term impacts on child growth and development.

**Methods:**

This study has used a community-based, cluster-randomized controlled trial with a superiority design and 2 parallel treatment arms. It is assessing the impact of peer counseling, starting in late pregnancy up to 1 year after delivery, on child feeding practices, growth, and development with follow-up until 48 months of age. The study site was Mirpur, a densely populated area in Dhaka. Using satellite maps and geographic information system mapping, we constructed 36 clusters with an average population of 5000 people. We recruited pregnant women in the third trimester aged 16-40 years, with no more than 3 living children. Trained peer counselors visited women at home twice before delivery, 4 times in the first month, monthly from 2 to 6 months, and again at 9 and 12 months. Trained research assistants collected anthropometric measurements. The primary outcome will be differences in child stunting and mean length for age at 6, 12, 15, and 18 months. Secondary outcomes will be differences in the percentage of women exclusively breastfeeding in the mean duration of any breastfeeding and in the percentage of children at 6 and 9 months of age who receive solid, semisolid, or soft foods; and the percentage of children consuming foods from 4 or more food groups at 9, 12, 15, and 18 months. We will assess the mean cognitive function scores from the Ages and Stages Questionnaire (9 and 18 months) and Bayley tests (24 and 36 months).

**Results:**

We identified 65,535 people in mapped residences, from which we defined 36 clusters and randomly allocated them equally to intervention or control groups stratified by cluster socioeconomic status. From July 2011 to May 2013, we identified 1056 pregnant women and 993 births in the intervention group and 994 pregnancies and 890 births in the control group. At 18 months, 692 children remained in the intervention group and 551 in the control group. From January 2015 to February 2017, we conducted the long-term follow-up of the cohort. We have now completed the data collection and processing and have started analyses.

**Conclusions:**

This study will help fill the evidence gap about the short- and long-term impact of peer counseling on improving infant feeding, preventing childhood undernutrition, and enhancing child cognitive development.

**Trial Registration:**

ClinicalTrials.gov NCT01333995; https://clinicaltrials.gov/ct2/show/NCT01333995

**International Registered Report Identifier (IRRID):**

DERR1-10.2196/31475

## Introduction

Although progress has been made in reducing child undernutrition [1], it remains a highly prevalent condition in low- and middle-income countries [2]. South Asia has a major portion of the global burden of childhood undernutrition, with an estimated 59 million (or 33.3%) of children under 5 years living with stunted growth in this region [3]. Undernutrition contributes to 45% of deaths in children under 5 years old [4], and effective interventions are needed to reduce its prevalence, particularly in countries with high prevalence. In Bangladesh, child undernutrition is an important public health problem, with 36% of children younger than 5 years being stunted, 33% being underweight, and 14% being wasted in 2014 [5].

Suboptimal breastfeeding increases the risks of childhood morbidity, which potentially contributes to the development of undernutrition and mortality, particularly in the first 2 years of life [2]. The World Health Organization (WHO) recommends exclusively breastfeeding children until 6 months of age, and after that, continuing breastfeeding with appropriately diverse and frequent complementary foods [6,7]. The complementary feeding period from 6 to 24 months also provides a window of opportunity for preventing undernutrition [8] as most postnatal growth faltering occurs during the first 2 years of life. Studies have shown that even with optimum breastfeeding, growth faltering will occur if children do not receive an adequate quantity and quality of complementary foods after 6 months [9].

A study from 2016 has shown that most stunting occurs in the first 2 years [10]. This growth retardation happens because children have a higher demand for nutrients and a higher rate of infectious diseases such as diarrhea, which adversely affects growth and nutritional status [11]. A lack of diversity of foods given to young children also contributes to undernutrition. For example, animal-source foods are an important source of protein and micronutrients, such as zinc, and low intakes of these foods are associated with stunting in children [8]. Improved breastfeeding practices and more diverse foods, and increased frequency of complementary feeds can help protect infants from childhood undernutrition and improve their developmental potential.

Growth faltering, in turn, has effects on a child's developmental potential [12,13]. The South Asian region also harbors a very high proportion of children with low cognitive and socioemotional Early Childhood Development Index scores [14]. Few studies have examined the relationship between undernutrition and cognitive development in Bangladeshi children. The evidence available comes mainly from 5 trials of psychosocial stimulation [15-19]. In all these studies, malnourished children had very low developmental scores, which were significantly lower than those of better-nourished children. In micronutrient supplementation trials, children's height-for-age and weight-for-age *z* scores were significantly correlated with their developmental levels, indicating a close association of nutritional status and child development [20-25].

A systematic review and meta-analysis of 110 studies for breastfeeding promotion found statistically significant increases in exclusive breastfeeding (EBF) rates: 43% at day 1, 30% at <1 month, and 90% at 1-5 months. Rates of “no breastfeeding” reduced by 32% at 1 day, 30% at <1 month, and 18% at 1-5 months [26]. An established approach to promoting appropriate breastfeeding practices in low- and middle-income countries involves local peer counselors providing information and support to mothers feeding young children [27,28]. A systematic review showed that in low- and middle-income countries, compared to usual care, community-based peer support increased EBF in the first 6 months and initiation of breastfeeding within the first hour of life, and decreased the risk of prelacteal feeding [29]. There is research demonstrating the feasibility of implementing a peer-counseling intervention in urban Bangladesh with evidence of changed feeding behaviors [28]. To date, there have been few reports of using peer counseling to educate, support, and build a mother's skills regarding both breastfeeding and appropriate complementary feeding. A before-and-after study conducted in a lower socioeconomic population in the Lalitpur district of Uttar Pradesh, India, suggests that peer counselors can improve infant feeding practices by increasing EBF and appropriate complementary feeds [30]. However, there is a need to examine the impact of peer counseling interventions on long-term child undernutrition, growth, and development.

We used the Standard Protocol Items: Recommendations for Interventional Trials (SPIRIT) guideline [31] to prepare this protocol. It was peer reviewed by the Australian National Health and Medical Research Council (NHMRC) grant review panels in 2009 (GNT63329) and 2013 (GNT1071005) and by the Research Review Committee of the International Centre for Diarrheal Disease Research-Bangladesh (icddr,b). The University of Sydney Human Ethics committee and the Ethics Review Committee of ICDDR-B approved the protocols. This report combines the 2 approved protocols.

We aim to implement a community-based, cluster -randomized controlled trial to collect evidence of whether peer counseling of women to promote appropriate infant feeding can improve breastfeeding, complementary feeding, and child growth, and thus reduce the prevalence of stunting (low height-for-age) in their children up to 18 months of age. In addition, the proposed study will examine the long-term impact of peer counseling for appropriate infant feeding on child anthropometry and cognitive development at 36 and 48 months.

Our primary hypothesis is that in a community-based, cluster-randomized controlled trial of peer counseling to support infant feeding starting from the third trimester of pregnancy to 1 year after delivery will reduce the prevalence of stunting (length-for-age < –2 z score) in children at 18 months of age by 10% compared to the control group with no peer counseling.

The short-term outcomes (secondary hypotheses) are the following:

1. The percentage of children with low length-for-age, low weight-for-height, and low weight-for-age between 6 and 18 months of age will be lower in the peer counseling group than in the control group.

2. The mean length-for-age, weight-for-height, and weight-for-age in children between 6 and 18 months of age will be higher in the peer counseling group than in the control group.

3. The children's height and weight velocity from birth to 18 months of age will be higher in the peer counseling group than in the control group.

4. The percentage of women EBF (breast milk and no other foods or milk-based liquids) their infants at 3 and 6 months will be higher in the peer counseling group than in the control group.

5. The mean duration of any breastfeeding will be longer in the peer counseling group than in the control group.

6. The percentage of women bottle feeding (any liquid or semisolid food from a bottle with a nipple or teat) their infants at 12 months will be lower in the peer counseling group than in the control group.

7.The percentage of children at 6 and 9 months of age who receive solid, semisolid, or soft foods will be higher in the peer counseling group than in the control group.

8. The percentage of children consuming >4 food groups at 9, 12, 15, and 18 months will be higher in the peer counseling group than in the control group.

9. Mean intake of children’s food energy, protein, carbohydrate, fat, and selected micronutrients (eg, zinc, iron, phytate vitamin A) from complementary feeds at 9, 12, 15, and 18 months will increase more in the peer counseling group than in the control group.

10. The mean days ill with diarrhea, acute respiratory illness, and fever at each monthly recall period from 1 to 18 months will be lower in the peer counseling group than in the control group.

The longer-term outcomes (secondary hypotheses) are the following:

1. The percentage of children with low length-for-age, low weight-for-length, and low weight-for-age in children between 18 and 48 months of age will be lower in the peer counseling group than in the control group.

2. The mean length-for-age, weight-for-length, and weight-for-age of children between 18 and 48 months of age will be higher in the peer counseling group than in the control group.

3. The children's length and weight velocity from 18 to 48 months of age will be higher in the peer counseling group than in the control group.

4. Mean cognitive, language, and motor composite scores as measured by the Ages and Stages Questionnaire and Bayley Scales of Infant and Toddler Development at 48 months of age will be higher in the peer counseling group than in the control group.

## Methods

### Study Design

**Figure 1:**
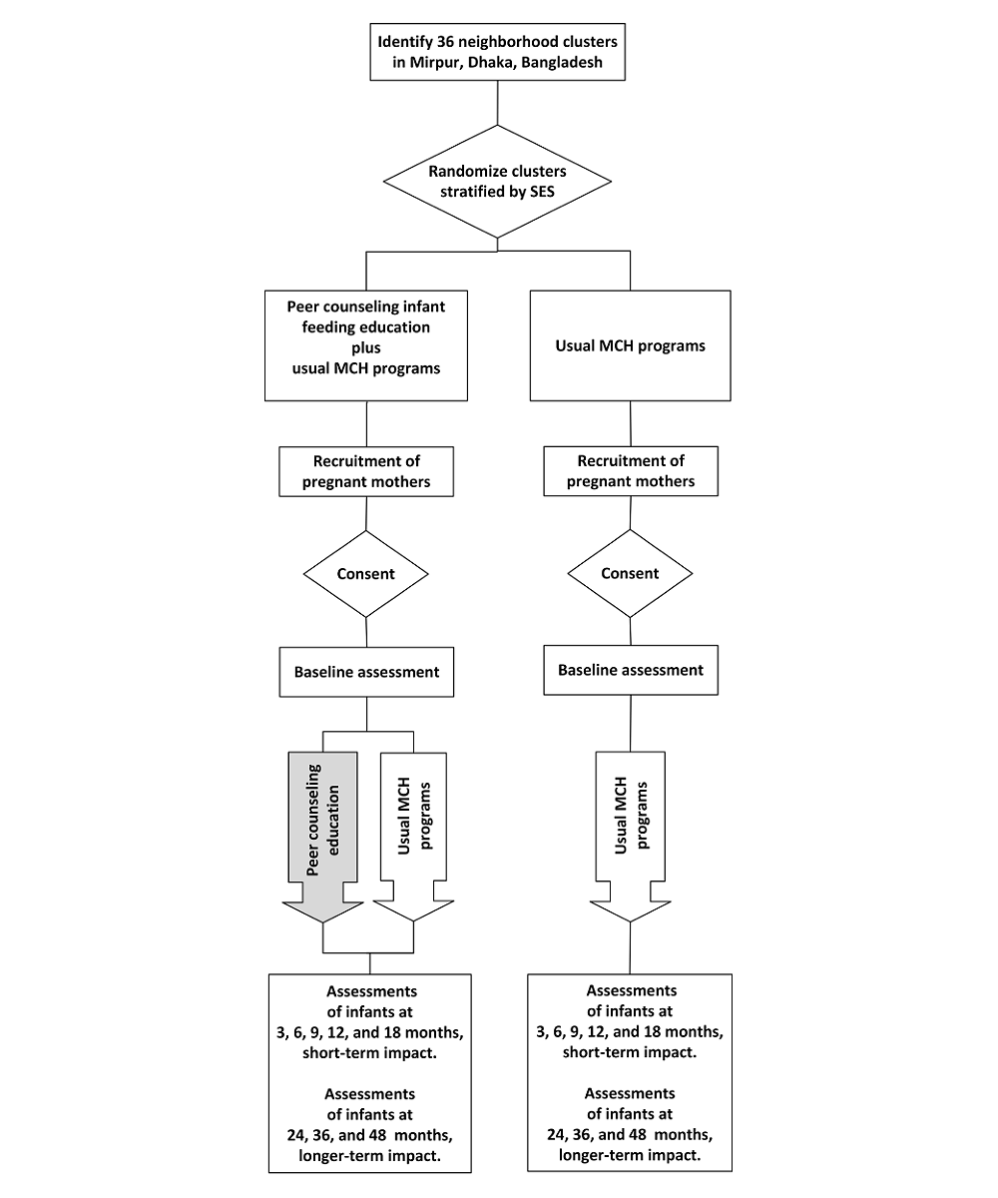
The Shishu Pushti study design. MCH: mother and child health; SES: socioeconomic status.

The study is a community-based, cluster-randomized controlled trial with a superiority design and 2 parallel treatment arms with a 1:1 allocation ratio. It examines the impact of a peer-counseling infant-feeding education program starting in the third trimester of pregnancy to 1 year after delivery on child feeding practices, growth, and development (Figure 1). We followed the cohort of the mother-child dyads from recruitment until the children were 48 months of age.

### Study Setting

We conducted the trial in Mirpur, an area of approximately 39 square kilometers located in the northwest of Dhaka City, Bangladesh. It has 16 wards (administrative regions), of which 4 wards were selected for the trial where there were no other major health or nutrition studies ongoing. Mirpur is a middle-to-lower socioeconomic area in Dhaka, but it includes very disadvantaged slum areas.

### Identification of Clusters

**Figure 2:**
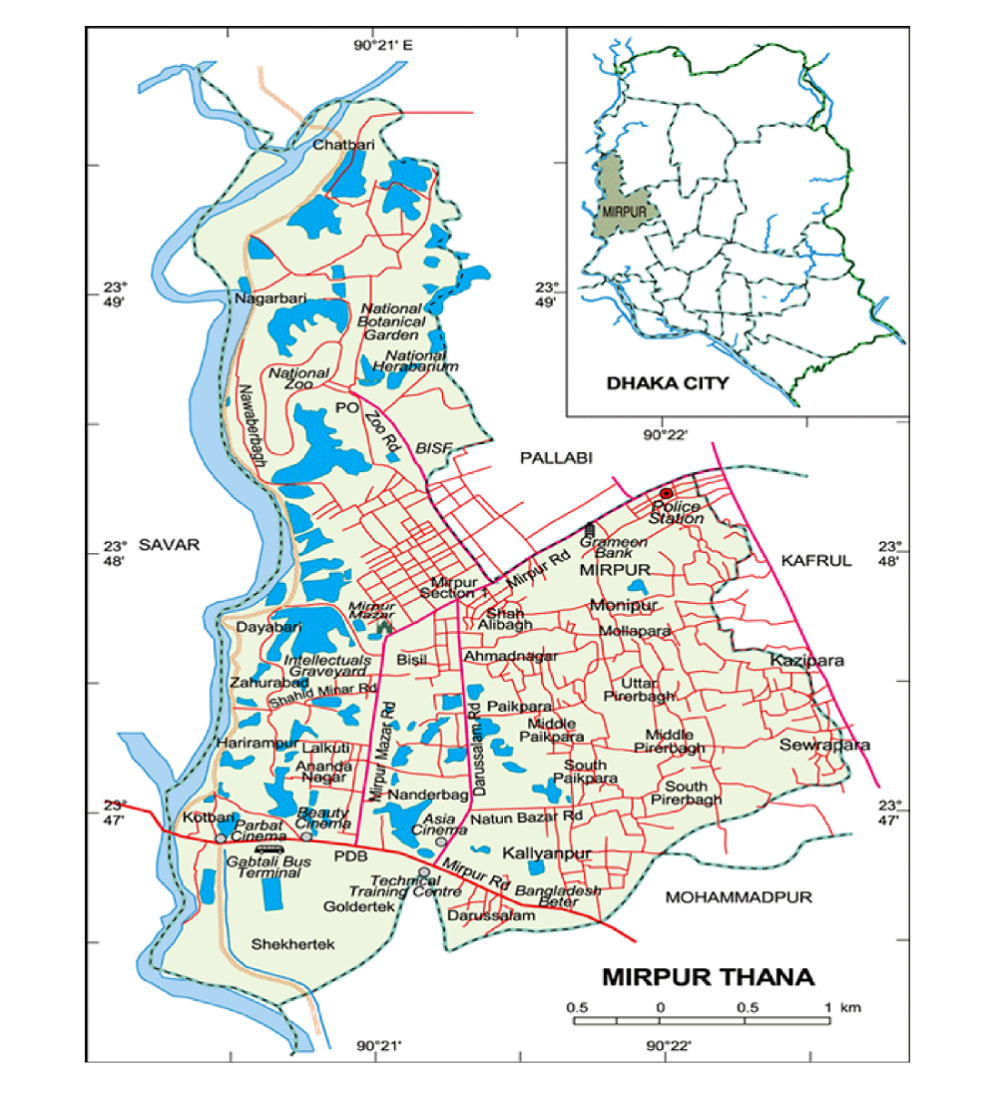
Map of the study site [32].

The study site is a very densely populated and includes areas of slum housing where there are no established addresses and houses are very close to each other against each other along narrow paths. In this setting, there were no administrative or natural boundaries to define clusters. For the trial, we used satellite maps of the study areas in Mirpur to define clusters with buffer areas. We digitalized all structures, buildings, roads, and water bodies from satellite maps of the study area and gave each structure an identification number. Our field census team visited all the structures and buildings in the study area and identified if it was a residence or was used for other purposes. After obtaining written informed consent, we conducted a brief household census and screened for pregnant women in all residential buildings. We used the census data to create a population database linked to the mapped structures. Using geographic information system mapping techniques, we constructed 36 clusters from the population maps to cover an average population of 5000 people with a surrounding 200-meter buffer zone between clusters. We classified each cluster as low socioeconomic status or not based on the census data. Figure 2 shows a map of the study site.

### Assignment of Treatments

We randomly allocated the 36 clusters in an equal ratio to intervention or control groups but with stratification by socioeconomic status of the clusters. We generated the random allocation sequence using SAS software (SAS Institute).

### Recruitment, Inclusion and Exclusion Criteria, and Consent of Mothers and Their Families

We visited the pregnant women identified in the census, and recruited eligible women in their third trimester of pregnancy into the trial. We updated the census data, including the pregnancy screening, every 3 months to identify and recruit potentially eligible women once they reached their third trimester. We included pregnant women who were 16 to 40 years of age and who had no more than 3 living children. We excluded women who planned to migrate from the Mirpur area after delivery and women with medical records of heart disease, tuberculosis, gestational diabetes, or eclampsia in previous pregnancies, from the trial. In addition, we excluded mother-infant pairs where the child had congenital abnormalities, had a very low birth weight below 1.5 kg, or were admitted to a neonatal intensive care unit. We used the same approach to recruitment in both intervention and control clusters. Trained project staff obtained written informed consent from the eligible women and their husbands. Based on experience with similar projects in Bangladesh [33,34], we expected at least 95% of the mothers would consent to participate. We anticipated excluding up to 30% of the women mainly related to their desire to migrate back to their home village after the delivery [34].

### Intervention Plan

We selected the trial intervention due to its feasibility, previous testing in urban populations in Dhaka [34], and likely future sustainability. We expect the proposed individual peer counseling education will have sufficient intensity to alter infant and young child feeding practices and improve young children's growth. After 1 year of preparation for the trial, we delivered the interventions to recruited pregnant women until their children reached 1 year of age.

This approach promoted appropriate infant and young child feeding through a program of home-based peer counseling by trained local women from the mothers' community. The peer counselors reached mothers who delivered at home and allowed the messages to reach other key family members who played a role in supporting breastfeeding and influenced the choice of foods for the infant. The main messages encouraged early initiation of breastfeeding, promoting EBF during the first 6 months of life, promoting appropriate timing of the introduction of complementary feeds, and ensuring an adequate frequency of feeds and diversity of foods used in their preparation.

#### Selection and Training of Peer Counselors

Women with personal breastfeeding experience and at least 6 years of schooling who resided in the same area and who were motivated to work to become peer counselors were selected. We trained the peer counselors using the joint WHO/UNICEF (United Nations International Children's Emergency Fund) breastfeeding counseling course adapted to the local language and culture and validated in a previous study [34]. They were trained for 40 hours (4 hours daily for 10 days). Counseling skills were taught mainly by demonstrations and role-play. The topics included listening to mothers, learning about their difficulties, assessing the position and attachment of infants during breastfeeding, building mother's confidence, giving support, and providing relevant information and practical help when required. During the course, the trainee counselors practiced antenatal and postpartum counseling at the field site with pregnant women, mothers with newborns, and infants aged 1-12 months. We trained counselors in how to use locally available foods for complementary feeding of infants and young children and how best to demonstrate these food preparation skills to mothers.

We anticipated that each peer counselor would support up to approximately 50-60 mothers and thus provide support to about 1000 women receiving the intervention. We recruited and trained 18 peer counselors (one in each cluster). Senior infant feeding counselors monitored the performance of the peer counselors at least 4 times during the study.

#### Counseling Schedules

We scheduled 13 visits by the peer counselors: 2 before delivery, 4 during the first month, 5 monthly visits from age 2 to 6 months, and 3 monthly visits at age 9 and 12 months. The counselors were free to make additional visits if the mother's circumstances required them. The counseling took place at home to include key family members (eg, mother-in-law and fathers) in the counseling sessions. The duration of each visit was from 20 to 40 minutes.

#### Antenatal Visits

During the 2 antenatal visits, peer counselors prepared and informed the mothers and other family members who supported the mothers at delivery about the importance of holding the infant within a few minutes of delivery and breastfeeding initiation within 1 hour of delivery. They discouraged prelacteal feeds and encouraged the mothers to eat more foods and rest during the third trimester. These meetings also covered problems with breastfeeding that the mothers might have encountered and provided strategies to deal with them.

#### Visits in the First Month of Life

The mothers were contacted 4 times by the peer counselors (within 48 hours of delivery, at 5-7 days, at 10-14 days, and at 24-28 days). During these visits, EBF was encouraged, and any barriers to EBF, such as sore nipples, problems with attachment and position of the baby during feeds, family pressure to introduce other foods, and mothers' doubts about the adequacy of their breastmilk, were resolved. If the counselor could not resolve these issues, the mothers were referred to the senior infant-feeding counselors.

#### Visits From 2 to 6 Months of Life

The mothers were contacted monthly by the peer counselors, who addressed any specific problems and continued to provide support for EBF, especially how to deal with family pressures, the introduction of other foods, and concerns about the adequacy of the infants’ growth. From 5 months of age, specific messages covered the importance of complementary feeding and demonstrations and preparation of complementary foods from homemade, regular family food. Mothers were given measuring bowls (250 ml) and spoons to promote the age-specific frequency of complementary feeding. According to the UNICEF guidelines, mothers received hands-on training on the frequency and amount of complementary feeding [35]. For the average healthy breastfed infant, meals of complementary food are needed 2-3 times (half a bowl of 250 ml) per day at 6-8 months of age and 3-4 times per day at 9-11 months and 12-23 months of age with additional nutritious snacks. Mothers were discouraged from using bottles for complementary feeding.

#### Visits From 6 to 12 Months of Life

The mothers were contacted twice at 3-month intervals by the peer counselors. They were encouraged to continue breastfeeding and supported to give an adequate frequency of complementary feeds and appropriate diversity of foods. There were further demonstrations on the preparation of complementary feeds as needed.

#### Management of Interventions

We recruited 3 breastfeeding counselors who trained the peer counselors under the supervision of 1 of the investigators (RH) and the research investigator (GA). The breastfeeding counselor provided technical support to the peer counselors and helped them resolve problems encountered during the implementation of the trial interventions by regular meetings with them in the field. The research officer supervised, monitored, and provided the necessary support for troubleshooting for breastfeeding complementary feeding. To facilitate the overall implementation process, we established an advisory committee consisting of local health officers, local government officials, representatives of mothers' groups from the community, representatives of the Bangladesh Breastfeeding Foundation, other relevant nongovernmental organizations, and project staff.

### Evaluation Plan

**Figure 3:**
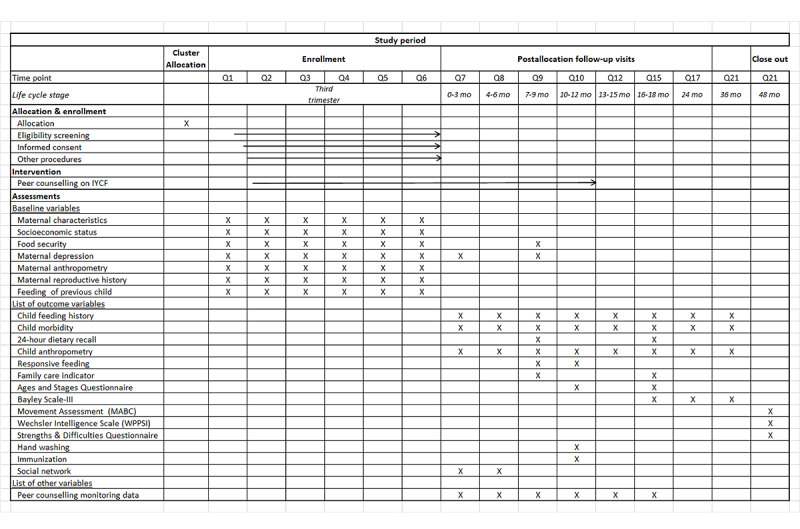
Schedule of enrollment, interventions, and assessments. IYCF: Infant and Young Child Feeding.

We applied a range of quantitative data collection methods to assess the primary and secondary outcomes, including pretested structured questionnaires and anthropometric measurements. Figure 3 presents a timeline depicting the schedule of data collection.

### Primary Outcomes to be Measured

The primary outcome measured was the differences in the percentage of stunted infants (length-for-age <–2 *z* score) of children at 6, 12, 15, and 18 months. We also measured changes in mean length-for-age *z* scores from birth until 48 months.

### Secondary Outcomes to be Measured

We measured changes in the percentage of women EBF (breast milk and no other foods or milk-based liquids) their infants from 1 to 6 months. We also measured changes in the mean duration of any breastfeeding. We calculated the changes in the percentage of children at 6 and 9 months of age who received solid, semisolid, or soft foods; and the percentage of children consuming foods from 4 or more food groups at 9, 12, 15, and 18 months. We assessed bottle feeding (any liquid or semisolid food from a bottle with a nipple or teat) in infants at 7, 9, and 12 months. We measured the changes in mean cognitive function scores with the Ages and Stages Questionnaire (9 and 18 months) and Bayley tests (24 and 36 months).

### Measurements

#### Anthropometry

Trained research assistants collected the anthropometric measurements (weight and height) using established methods [36] and recorded these measurements on both the research instruments and an infant growth chart for the mother to keep. We standardized these measurements before and during the data collection. Anthropometry was collected soon after birth and every month up to 12 months, at 3-month intervals up to 18 months (15 and 18 months), and then at 24, 36, and 48 months. We used the 2006 WHO growth standards to construct anthropometric indices. We calculated the standard WHO-recommended indicators to assess stunting (height-for-age <–2 *z* score), wasting (weight-for-height <–2 *z* score), and being underweight (weight-for-age <–2 *z* score).

#### Infant Feeding Practices

We collected infant feeding data every month from birth to 6 months, and then at 3-month intervals between 6 and 18 months. We applied the standard Bangladesh Demographic Health Survey (DHS) questions about infant-feeding practices [37] and monitored these patterns at the 3 monthly data collection periods from birth until 18 months of age. These include questions about current breastfeeding status, current use of other liquids and solid foods, the timing of introduction of other liquids or solid foods, use of bottles for feeding, and information about who was providing advice about infant feeding among family and friends.

A senior research assistant monitored about 10% of the interviewer's scheduled visits. The supervisors checked questionnaires daily, and if the information was incomplete or not clear, they returned to the home on the next day to complete the data form. Senior research assistants verified the mother's reports of infant feeding practices through a 4-hour observation period in an unscheduled visit.

#### Dietary Intake

Trained interviewers collected 24-hour dietary recalls using standard methods starting at 6 months of age until 18 months. They recorded all the foods consumed 24 hours before the interview and measured the portion sizes in local utensils. They also recorded the recipes used to prepare foods, including the amounts of raw food used and the preparation methods. Research assistants analyzed and presented the nutrient intakes and food groups consumed. In a subsample of 10% of respondents, we took duplicate 24-hour recalls to assess within-person variability and to allow adjustment of the prevalence of low intake nutrients.

#### Birth Weight and Gestational Age

Trained field research assistants measured the birth weight of the neonate within 72 hours of birth. In the intervention clusters, the peer counselors provided delivery information to the research team in timely fashion. In the case of control clusters, we recruited volunteers responsible for the timely reporting of the delivery information.

#### Child Morbidity

The interviewers obtained illness histories, such as diarrhea, dysentery (blood and mucus), fever and cough, and ear infection (purulent discharge), every month until 12 months, and at 3-month intervals until 18 months using the 2-weeks' recall method. The questions asked were based on the standard DHS infant morbidity recall questions but were expanded to include questions about ear discharge.

Diarrhea was defined as an episode of the passage of 3 or more loose or watery stools within 24 hours. The presence of blood in the stool was defined as invasive diarrhea. When a single episode of diarrhea lasted for more than 2 weeks, it was be classified as persistent diarrhea. Acute respiratory illnesses were defined as an episode of cough with reported fast and rapid breathing or difficulty breathing with or without fever.

#### Child Development

We collected a variety of child development measures at 9, 18, 24, 36, and 48 months. The Ages and Stages Questionnaire [38] was used to collect data on problem-solving, socioemotional, communication, and fine and gross motor functions at 9 and 18 months of age. The Bayley Scales of Infant and Toddler development-III (Bayley-III) [39] was used to assess children's cognitive, language, and motor development at 24 and 36 months of age. The test is not standardized in Bangladesh but has been culturally adapted and used in several studies on Bangladeshi children [40,41]. The Wechsler Preschool and Primary Scale of Intelligence (WPPSI) [42] will be used on a subsample of children at 48 months to assess their full scale, verbal, and performance IQs. The children's behavior during the Bayley test was rated using 5 behavior ratings developed by Wolke et al [43], each of which has ratings from 1 to 9, with 1 being the poorest behavior and 9 being the best. We interviewed mothers on child behaviors using the Strengths and Difficulties Questionnaire [44] at 48 months after the WPPSI test.

The Movement Assessment Battery for Children [45] will be administered along with WPPSI at 48 months of age on the same subsample.

Information on quality of home stimulation was assessed by a family care indicators questionnaire [46] at 9, 18, and 24 months. An early childhood version of the home observation for measurement of the environment [47] was administered at 24 and 36 months to collect information on approaches that the family employs, such as availability of toys, books, musical instruments, play activities with the child, and other learning opportunities at home.

#### Maternal Depression

We assumed that the mother's mental well-being or depression condition could affect the child's nutrition and caring practices, which can mediate child development. We collected information regarding the mothers' mental status and depression conditions in the third trimester of pregnancy and at 1 and 10 months postpartum from all participants using the Center for Epidemiological Studies-Depression questionnaire [48].

Maternal IQ was measured using the Raven Coloured Progressive Matrices [49].

#### Socioeconomic Status

Information on religion, pregnant women's and her husband's levels of education, their occupations, number of family members, ownership of the number of dwelling rooms, household construction materials, toilet facilities, drinking water sources, household assets, and land ownership were collected as the key indicators of socioeconomic status.

#### Household Food Security

As household food security plays a vital role in complementary feeding practices and child nutrition, we assessed the family's food security from all participants (during enrolment and at 10 months).

#### Information on Social Network

Family members, friends, neighbors, peers, and people around caregivers play important roles in feeding and caring for young infants. Therefore, we collected information on the social networks of caregivers at different times of follow-up from 100 families in 4 clusters.

### Schedule of Data Collection

We carried out a pilot study in the first year of the trial in a single cluster to test the recruitment methods, implementation of the intervention, the evaluation of instruments, and field methods before commencing the trial.

For the assessment of trial outcomes, we collected basic sociodemographic information about the family and maternal characteristics in a baseline survey at enrolment. We recorded the details of the birth and pregnancy shortly after delivery. We assessed the trial outcomes by measurements at monthly intervals from birth until 12 months, at 3-month intervals until 18 months, and annually until 48 months. These included anthropometric measurements, the recording of dietary patterns, dietary intake, and morbidity. The schedule of data collection is given in Figure 3.

### Process Evaluation

We conducted a mixed methods process evaluation within the Mirpur Shishu Pushti Trial to answer the primary question of how and why or why no impacts were achieved [50,51]. The specific aims included the assessment of fidelity, dose, reach, and intensity of the peer counseling intervention; the response of the beneficiaries to the intervention; experience of the peer counselors of counseling the women; and the barriers and challenges encountered by the project staff to implement the trial.

We further aimed to synchronize the process evaluation data collection with the timeline of program implementation. Data collection has been planned to occur in 2 stages: (1) ongoing throughout the intervention delivery and (2) at a time point near the end of the intervention. This approach will enable us to use the process evaluation data for both providing feedback to keep the program on track (formative use) and to interpret and explain intervention outcomes (summative use) [52].

We collected the data for the process evaluation through regular project monitoring on the participation of mothers in peer counseling sessions, surveys of self-reported adherence to counseling guidelines by the peer counselors, unscheduled direct observations of the peer counseling at-home visits, in-depth interviews of mothers, semistructured interviews with the project managers (GA) and the project field staff, and focus group discussion with peer counselors. We collected the data in a purposively selected sample of intervention clusters to cover the variations within the households.

### Sampling Design and Sample Size

The sample size required was 1950 mother-infant dyads (975 in each treatment group) from 50 clusters with 39 mother-infant dyads per community cluster recruited over 3 months. The sample size calculation was estimated with the following assumptions: Each community cluster had an average population of 70,000 and an expected crude birth rate of 4.3 per 1000 total population over 6 months (based on the crude birth rate for urban Bangladesh of 25.8/1000 population over 36 months from the 2004 Bangladesh DHS]) thus giving an average expected number of births of 150 over 3 months per cluster. Previous research [34] indicated that appropriately 33% of pregnant women returned to their home village following delivery, leaving an expected number of eligible births of 200 over 6 months per cluster. We anticipated having 39 mother-infant dyads per cluster, assuming 95% participation but 22% loss to follow-up based on earlier research [34] from the approximately 200 mother-infant dyads available in each cluster over 6 months. We also assumed 90% power and a 5% two-sided α and intracluster correlation coefficient of 0.015 (based on analyses of the child anthropometric measurements from the 2004 Bangladesh DHS survey data for urban child populations [53]). We also expect the difference in the prevalence of stunting between the treatment groups of 10% (35% in control to 25% in the intervention group), which is similar to the change reported in an earlier education intervention for young child feeding in Peru [54]. 

### Statistical Analyses

Data analysis will be by intention to treat. Analyses will be conducted at the mother-infant dyad level but will be adjusted for the community-cluster randomization [55]. The primary analyses will compare the prevalence of stunting (length-for-age < –2 *z* score) in children at 18 months of age using Pearson chi-square tests and 95% CIs for the group difference, with adjustments for clustering. We will report the results for 2-sided 5% tests.

Secondary analyses will examine each outcome variable (length-for-age, percentage EBF, duration of breastfeeding, percentage doing bottle feeding, percentage giving complementary feeds, percentage receiving diverse food groups, and mean nutrient intakes), taking account of the repeated measurements within children by using separate mixed models. We will use linear mixed models for the continuous outcomes (eg, height for age *z* score, duration of breastfeeding, Ages and Stages Questionnaire, Bayley, and Wechsler scales) and generalized linear mixed models for noncontinuous outcomes (eg, logistic mixed models for binary outcomes, such as percentage stunted or percentage exclusively breastfeeding). Models will include the treatment group as a fixed effect and infants as a random effect to account for the repeated measurements, and community cluster as a random effect to account for the cluster effect. The models will evaluate the impact of the interventions over time by testing for an interaction between time and the intervention group.

We will conduct analyses to identify the baseline characteristics of the mother-infant dyads who may benefit most from the intervention. Model assumptions will be checked, and appropriate adjustments to the analysis will be made where necessary. We will use Stata software (Stata Corp) for all analyses, with the “mixed” command to fit linear mixed models, the “melogit” command to fit mixed-effects models for binary outcomes, and the “mepoisson” command to fit mixed-effects models for event count data.

### Research Ethics

We obtained ethical approval for the study from the research ethics committee of icddr,b (#PR-10001) and The University of Sydney (#12900). Trained field staff carefully explained the background and objectives of the study to the women and gave a written information sheet to all women contacted. We obtained written informed consent from those women who agreed to participate in the study. We will maintain the privacy, anonymity, and confidentiality of the information provided by respondents during all phases of the trial. We will store all information in an encrypted database with the participant's study identifier instead of with personal identifiers. Only the investigators and an authorized data management team will have access to collected data.

### Data Access

All data collected will be accessible by the study investigators, who will have the right to analyze and publish data. We will make the relevant anonymized individual-level data available upon reasonable request.

## Results

Using satellite maps, we digitized all structures in the study areas, and from a brief census of all mapped residences, we identified a population of 65,535 people. We created a population database linked to the map structure and applied this information to define 36 clusters with 500-meter buffer zones. We randomly allocated the 36 clusters in an equal ratio to intervention or control groups but stratified by the socioeconomic status of the clusters. We screened for pregnant women in all residences and identified 5377 eligible pregnant women.

From July 2011 to May 2013, we carried out a baseline survey. In the third trimester, we examined 2050 pregnant women, including 1056 women in the intervention group and 994 women in the control group. The women participating in the trial had 1883 birth outcomes, with 993 in the intervention group and 890 in the control group. The study followed up the women who delivered the infants until 18 months of life. In December 2012, we began assessing the original cohort and completed 18 months of study follow-up in October 2014 for participants. Among them, 692 in the intervention group and 551 in the control group remained in the study until 18 months follow-up, with 301dropouts in the intervention and 339 dropouts in the control group. We continued evaluating the cohort until the children were 36 months of age, with child growth and development outcomes measured at 24 and 36 months. The long-term follow-up began in January 2015 and ended in February 2017. We have now completed the data cleaning and processing and have started analyses of the data.

## Discussion

The 2008 Lancet Maternal and Child Undernutrition series called for high quality “how-to” research to become a priority [9,56]. It is now widely accepted that the lack of EBF and inappropriate complementary feeding are critical factors contributing to undernutrition, morbidity, and mortality in children. However, interventions to improve these critical factors have had mixed success. Ascertaining what works and what does not work is important for continuing progress on alleviating undernutrition in children. We describe the protocol and processes for implementing a randomized controlled trial in urban Dhaka, Bangladesh. The trial aims to test the efficacy of peer counseling to improve breastfeeding and complementary feeding practices and reduce undernutrition in children under 4 years of age. Breastfeeding is almost universal in Bangladesh, and the rates of EBF have increased in recent years; however, the prevalence of appropriate complementary feeding remains low. The latest available representative data (from 2017 to 2018) show that 65% of children aged under 6 months are exclusively breastfed, and only 34% of children aged 6 to 23 months are fed appropriately according to recommended infant and young child feeding practices [5,57]. Previous studies conducted in urban areas of Bangladesh using individual peer counseling have shown significant increases in EBF rates [34]. Our research will build on this work and evaluate extended peer counseling supporting both breastfeeding and complementary feeding. 

There are several limitations and challenges to the implementation of our study. One challenge will be recruiting peer counselors from a highly mobile urban mobile population and working effectively in urban slum communities. Another challenge will be training peer counselors drawn from the same disadvantaged neighborhoods to provide support to mothers and information about appropriate breastfeeding and complementary feeding. Recruiting and retaining working women with many competing demands will also be a major challenge, potentially contributing to loss to follow-up in the trial.

There is an urgent need to develop and disseminate effective interventions to improve complementary feeding, and this project will help resolve this information gap. There have been no interventions that include peer counseling to improve infant feeding practices implemented over the critical window of the first 1000 days and beyond to 36 months of life. This study will help fill the evidence gap regarding the long-term impact of peer counseling on improving infant feeding, preventing childhood undernutrition, and enhancing child cognitive development. The intensity and duration of this intervention are likely to result in a reduced rate of undernutrition. They can be adapted for various contexts and used by public health planners in other developing countries. The publications expected to arise from this research will contribute substantially to the evidence base. They will help with the development of public health nutrition policies for children in South Asia.

## Appendix

Multimedia Appendix 1 Peer-reviewer report from the National Health and Medical Research Council of Australia, and International Centre for Diarrhoeal Disease Research, Bangladesh.

## Abbreviations

DHS: Demographic Health Survey
EBF: exclusive breastfeeding
icddr,b: International Centre for Diarrhoeal Disease Research, Bangladesh
NHMRC: National Health and Medical Research Council
SPIRIT: Standard Protocol Items: Recommendations for Interventional Trial
UNICEF: The United Nations Children's Emergency Fund
WHO: World Health Organization
WPPSI: Wechsler Preschool and Primary Scale of Intelligence
