# Supplementary material for: The Shishu Pushti Trial–Extended Peer Counseling for Improving Feeding Practices and Reducing Undernutrition in Children Aged 0-48 Months in Urban Bangladesh: Protocol for a Cluster-Randomized Controlled Trial
Source: JMIR Res Protoc. 2022 Feb 7;11(2):e31475. doi: 10.2196/31475 (PMC8861872; doi:10.2196/31475)
Supplement: Multimedia Appendix 1 [file resprot_v11i2e31475_app1.pdf]

ONHMRC - GrantNet

ASSESSMENT REPORT FOR APPLICANT

Application Id: 633229 **Chief Investigator A:** Prof Michael A Dibley **Application Year :** 2009

Title: Peer counseling to improve feeding practices and reduce malnutrition in children 0-2 years in Bangladesh

Assessor 1

Scientific Quality

The investigators propose to conduct a cluster RCT in which 50 Mallahas (geographic zones) in Dhaka, Bangladesh, will be randomised to intervention or control status prior to recruitment of eligible women. The proposal replicates many aspects of the earlier trial, albeit with a larger sample, extended period of follow-up and a larger range of outcome measures. Recruitment procedures, sample size calculations, inclusion and exclusion criteria, estimates of time required to recruit the required sample size (n= 1950), details regarding training and ongoing supervision for the peer counsellors, the schedule of home visits, data collection and quality control procedures are all clearly described in the proposal. A pilot study will be conducted in the first year of the study to assess recruitment, intervention and evaluation methods in a single cluster, and process evaluation will be undertaken to assess intervention fidelity, dose, reach and intensity. This will include unscheduled observations of peer counselling sessions and infant feeding over an extended time frame.

Strengths of the proposal include: a clearly defined intervention, prior research demonstrating feasibility of implementing the counselling intervention and willingness of study participants to participate in follow-up to 5 months pp; sample size calculations taking into account the cluster design, number of clusters, number of study participants per cluster and drawing on available data to estimate ICCs; analysis by intention to treat, taking into account the cluster randomisation method, repeated measures for secondary outcomes and impact of the intervention over time; and inclusion of economic evaluation to assess cost effectiveness.

Some issues require clarification. In the earlier trial women received an average of 15 visits from trained peer counsellors, while in the current study it is proposed to provide 13 visits over a longer time frame. What is the rationale for reducing the intensity of the intervention while extending the timeframe?

Based on the previous study the investigators estimate that 95% of women approached to participate will agree and 22% will be lost to follow-up. However in this study follow-up ceased at 5 months postpartum. Will the projected sample of n=1950 be adequate for assessment of the primary outcome at 18 months postpartum given that attrition is likely to be higher than 22%?

It is not clear how contamination between intervention and control areas will be avoided and/or assessed. Will it be possible to check for potential contamination effects during process evaluation?

Significance and Innovation

This carefully designed study aims to provide evidence regarding effective interventions to improve infant feeding and reduce child malnutrition in developing countries. The proposal builds on prior research by members of the research team demonstrating that trained peer counsellors providing home-based infant feeding education to mothers from late pregnancy to 5 months postpartum increased initiation and duration of exclusive breastfeeding in an urban population residing in Dhaka city, Bangladesh. This trial - published in the Lancet (2000) - did not include assessment of the impact of the intervention on infant growth, and follow-up was ceased at 5 months postpartum. In the current study, the investigators propose to recruit a larger sample, extend peer counselling to one year postpartum, and extend follow-up to 18 months postpartum, in order to assess the impact of peer counselling on a larger range of outcomes including: infant growth at 18 months postpartum and infant feeding practices at 3, 6, 9 and 12 months. Poor nutritional status of children in developing countries is recognised as a key public health problem contributing to the global burden of disease, and has been identified as a core concern in Millennium Development Goals. The proposed study will provide stronger evidence regarding the effectiveness of a home based peer counselling educational intervention in improving child feeding practices, improving child growth and reducing malnutrition in developing countries. The outcomes of the trial are likely to be of interest to public health planners and policy makers in developing countries, especially in the South Asian region, and assuming that the trial is well-conducted, results are likely to be published in high impact journals.

Track Record in Relation to Opportunity

The CIA is a nutritional epidemiologist with a strong track record of research in developing countries. He has received research grant income of >$2.25m from a range of international funding agencies including the World Bank, Nestle Foundation and UNICEF and has conducted technical consultancies for a broad range of agencies in South East and East Asia. He has published 38 peer reviewed papers in the past 5 years, including a co-authored paper in the BMJ and other papers in specialist journals in his field. While much of his research has been based in Asia, he holds current appointments at the University of Sydney, and conjoint appointment at the University of Newcastle and Jiaotong University in China.

The CIA is supported by CIs with expertise in nutritional epidemiology (CIB, CIC, CIE); international health (CIB, CIE), statistics (CID), and health promotion/educational interventions (CIB, CIE). CIs B, C, D and E are members of the South Asian Infant Feeding Research Network.

The CIB - who currently works at the International Centre for Diarrhoeal Diseases Research in Bangladesh - has attracted competitive funding from agencies such as the World Bank and UNICEF totalling US$2.5m, and has published 8 peer reviewed publications in the past 5 years. He is a co-author of the trial published in the Lancet on which the current proposal is based.

The CIC and CID are early career researchers with sound track records relative to opportunity. The CIC was project co-ordinator for a large clinical randomised trial - the Child Asthma Prevention Study - in Sydney from 1997 to 2007 and has 15 peer reviewed publications over the past 5 years, including 6 as first author. The CID is a statistician with experience in analysis of infant feeding data using multi-level modelling, and 15 peer reviewed publications. The CIE is a senior scientist based at the International Centre for Diarrhoeal Diseases Research in Bangladesh with a research interest in nutritional interventions and infant feeding.

All of the investigators propose to contribute substantive time fractions to leadership of this project, commensurate with the scope and complexity of the proposal. Roles and responsibilities are also clearly defined, with provision for the CIC (100% time fraction in years 1 & 2) to make regular field trips to Bangladesh. The CIB (50% time fraction) will lead the field work supported by CIE (both based in Bangladesh).

Budget

Salaries are requested for three CIs and several project staff:

CIB (PSP5, time fraction 50%) and CIE (PSP 5, time fraction 10%) have overlapping responsibility for recruitment and supervision of field staff, liaison with local government and health department, monitoring training on peer counsellors, and contributing to analysis plans and writing up results. Both of these investigators are senior scientists based in Dhaka, Bangladesh

CIC (PSP4, time fraction 100% in years 1-2, 50% in yr3, 25% in yr 4) who will co-ordinate development and pilot testing of study instruments,monitoring of the intervention through site visits, and contribute to data analysis. This investigator is based in Australia and will make several extended field trips to Dhaka.

Salary requests for project staff include: a full time project manager (PSP2); two Infant Feeding Counsellors responsible for training and monitoring work of peer counsellors (funded by one PSP4); six Senior Research Assistants to undertake data collection (funded by one PSP4); a data manager (PSP4); and three technical staff (at PSP1) contributing to data collection.

Direct research costs include: project set up costs ($85,000); honoraria for peer counsellors at US$50 per month ($285,000); data entry costs ($40,000) and costs of international travel for the CIs ($85,000).

Salary requests for the CIB and CIE have been inflated to reflect the actual salaries of these investigators which is not allowable under NHMRC funding rules. There is also substantial overlap in the roles of these CIs. Could the investigators provide further justification of the time fractions requested?

Salary requests for research staff are justified by the scale of this project (n=1950), frequent follow-up (3,6,9,12,15 and 18 months pp), range of measures (including anthropometric measures, infant feeding and dietary intake), monitoring of 10% of interviews by CIs or senior RAs, unscheduled 4 hour observation visits, and process evaluation to assess intervention fidelity, dose, reach and intensity. However, the budget for this trial is substantial and it could be argued that costs might be reduced by a less frequent follow-up regimen. Could the investigators comment on their rationale for follow-up at three monthly intervals and what cost savings and trade-offs would be involved in reducing the frequency of follow-up, for example by omitting the 9 month and/or 15 month follow-up/s?

There is also a need for more detailed justification of requests for project set up costs ($85,000) and international travel, in particular the request for CIB and CIE to visit Australia in year 4.

Questions for the Applicant

What basis do the investigators have for assuming that 13 visits will be sufficient to achieve the desired outcomes, given that the earlier trial, on which this study is based, involved 15 visits to women over a shorter time frame?

Will the projected sample of n=1950 be adequate for assessment of the primary outcome at 18 months postpartum given that attrition is likely to be higher than the projected 22%?

Has consideration been given to strategies to reduce the likelihood of greater attrition in the control arm of the trial (as occurred in the earlier trial)?

Will it be possible to check for potential contamination effects during process evaluation?

Could the investigators comment on their rationale for follow-up at three monthly intervals and what cost savings and trade-offs would be involved in reducing the frequency of follow-up, for example by omitting the 9 month and/or 15 month follow-up/s?

Could the investigators provide more information regarding the CIs experience leading large scale epidemiological studies, especially studies that have involved leadership of large multidisciplinary teams, and development of relationships with local NGOs and other agencies/stakeholders?

A large amount of data will be generated by the frequent follow-up of over 1900 mother-infant pairs. Who will be taking responsibility for design, implementation and maintenance of data management and quality control systems for the study?

Could the investigators please provide further justification of the time fractions requested for CIB and CIE, and more detailed justification of requests for project set up costs ($85,000) and international travel, in particular the request for CIB and CIE to visit Australia in year 4?

Additional Questions for the Applicant/General Comments

Application Id: 633229 **Chief Investigator A:** Prof Michael A Dibley **Application Year :** 2009

Assessor 2

Scientific Quality

I perceive that there is a basic flaw in the intervention because the complementary foods promoted as part of the intervention do not appear to offer an increase in the quality and quantity of bioavailable micronutrients needed to support growth and minimize infection. It would therefore be important to have some pilot data that the proposed intervention of peer counseling actually improves the micro nutrient intake or status of children in this setting before a major roll-out of the proposed clinical trial. This is especially so as the counseling schedule is very heavy in early infancy (with breastfeeding support) and there are very few counseling visits during the period where complementary foods are becoming a major source of nutritional intake (one visit at 9 months and the other at 12 months).

Some more information on the monitoring of the peer counselors and the quality of the advice would be useful.

Dietary intake will be monitored by 24 hour recall. It seems that this will be used as a proxy of the intake of individuals whereas this methodology is designed to give an estimate of the mean/median intake of groups or populations. Please clarify how these data will be used.

It was a surprise to me that there was no planned monitoring of adverse events in the trial. Will deaths and serous illnesses be documents? Days of fever? Days of bloody diarrhea? Days of antibiotics? It would seem that these are important outcomes for such a study

The grant seemed to be poorly referenced

The competitive position of the group was not clear. It would be useful to know how this group fits in with other studies that are planned or ongoing by other major groups in international health that work is South Asia (for example the John Hopkins group and the Emory group).

Some detailed information of the data management systems to be employed in the trial would be helpful.

Significance and Innovation

The investigative team highlight that there is a significant problem of malnutrition of young children in south Asia. This is clearly a major issue of great public health importance because of the health, economic and societal burden, although this argument is not well developed in the application.

The innovative aspect of this application is the extension of a peer-counseling approach that was used to promote appropriate breastfeeding practices to also promoting more appropriate feeding practices associated with complementary feeding (of solid foods). However, the investigators have not discussed the fact that one of the main nutritional issues in growth faltering of young children in developing or emerging economies is that the complementary foods are not only energy poor but they are also micronutrient poor and cannot provide the necessary micronutrients (such as iron and zinc) that are necessary at a time when breast milk alone is no longer adequate. The application suggests the addition of soybean oil (as energy from fat) to complementary foods but does not address the micronutrient issue.

Track Record in Relation to Opportunity

The group is qualified to do the study. They appear to have all the necessary political and on the ground connections.
CIA has relevant publications in appropriate journals for this field but the citation rate is very low.
The output of CIB is very modest
The publication output of CIC, CID and CIE is competent but the citation rate is low

Budget

The budget includes the salaries of CIB, CIC and CIE.
The budget seems appropriate for the level and extent of the proposed intervention and follow up

Questions for the Applicant

Please justify the lack of micronutrient intervention

Please comment on the feasibility of the peer counseling approach as a strategy to improve the quality of complementary feeding without specific micronutrient supplementation

Please comment on the approach to only increase dietary fat in traditional complementary foods

Please justify the choice of dietary assessment methods and the appropriateness for this study

Please provide some detail on the competitive position of your group relative to other groups in international nutrition/health working in South Asia

Please justify why adverse events are not being monitored

Please expand on the data management systems to support the integrity of the data Bangladesh and the transfer to Australia

Additional Questions for the Applicant/General Comments

Assessor 3

Scientific Quality

The design is very clear, with the sampling unit well justified. The descriptiion of the intervention is very clear, and the sample size is adequate. However, the recruitment method the the inevstigators will use has not been tested and validated. How confident are the investigators that they can recruit the required sample size? It cannot be assumed that pregnant mothers would be willing to participate in such trial. How is attrition going to be prevented or addressed? No acknowledgement is made of pregnant mothers' sensitivity to intrusive breast feeding education (e.g. breastfeeding initiation), and no discussion on how this might be dealt with during peer counselling. Peer counsellors will be trained using the UNICEF module, but it is not clear how the effectiveness of the training per se will be evaluated. If the peer-counsellors do not grasp the training or are not competent, the whole program will fall apart and may fail. It is important that the peer counselling training is evaluated and peer counsellors' competency established at the formative phase of the program. these issues are not adequately addressed in the proposal.

Significance and Innovation

Although the planned research addresses issues of some importance to human health in developing countries, the significance and scope have not been articulated comprehensivley. There is no doubt that poor nutritional status of children is a major public health problem throughout the developing world and contribute significantly to child deaths. However, I question the focus on stunting (height-for-age) as the outcome measure. Even, when implementing WHO guidelines on the management of malnutrition in controlled enviornments such as refugee camps or among internally displaced perople, through summplementary feeding program, the impact of such programs on stunting is always minimal. But changes are likely to occur for wasting (weight-for-height z score) and in some cases for underweight (weight-for-age z scorre). Since this research will be imbedded in existing MCH programs, one would have expected underweight to be the outcome measure for measuring the program effectiveness. In addition, it is well known that the risk of malnutrition and failure to thrive within the first 3 years of life in many developing countries is due to co-morbidity mainly measles, interstinal parasites, diarrheal diseases, ARIs and malaria (if in endemic region). As the malnutrition-infection vicious circle becomes clear, the contribution of malnutrition to mortality become evident, but this poorly articulated in the background. Consequently, the focus on only breastfeeding is a bit misleading. The proposed research does not seem to consider commorbidity as confounding factors. It is not clear whether the research consider measles immunisation coverage, de-worming, diarrheal control etc. to be critical when measuring the effectiness of the proposed program as they are not included in the project evaluation framework. Finally, It could have prudent to consider all three anthropomtric outcomes: weight for age, heaight for age and weight for height, as to examine which one will reposend well to the proposed intervention

Track Record in Relation to Opportunity

CIA has a strong track record and high quality articles in the proposed area. He has mainly published in the area of the proposed research. However, CIB has only 8 articles and the quality of the publications is not strong (only one publication does seem to have an impact factor), CIC has a strong track record, but their publication does not seem to match the skill required for the proposed project. Given that the proposed program may require some persons with expertise in cultural competence,anthropology, economic evaluation, health literacy etc, it would have been prudent to have a CI with a strong track record in cultural competence, an anthropologist, health literacy specialist (given the peer counselling approach), and a specialist community engagement approaches and frameworks, a health economist. These skills are lacking in the team. It is not clear how the skill gap will be addressed

Application Id: 633229 **Chief Investigator A:** Prof Michael A Dibley **Application Year :** 2009

Budget

As indicated earlier, the budget is mainly salaries. Salaries are requested for CIB, CIC, and CIE plus various Personnel Support Packages. what is the role of CIs since most technical work is budgeted for through Personnel Support Packages? How are the CIs going to afford the skill gap (experts in cultural comptence, health literacy, community engagement specialist, economic evaluation etc.) given that the team make up represents a duplication of expertise rather than diverse but complementary expertise. For example, wouldn't one want to know whether the peer-counselling was cost-effective, hence requiring a health economist? The economic evaluation has not been budgeted for, and CIs do not seem to have expertise in cost-effectiveness analysis

Questions for the Applicant

Why the focus on stunting rather that underweight given that the program is being implemented through MCH? Who will carry out the economic evaluation? how is attrition going to be addressed?

Additional Questions for the Applicant/General Comments

The team make-up could have benefited from diverse but complementary expertise
